# How Many Participants? How Many Trials? Maximizing the Power of Reaction Time Studies

**DOI:** 10.3758/s13428-023-02155-9

**Published:** 2023-08-03

**Authors:** Jeff Miller

**Affiliations:** Department of Psychology, University of Otago, PO Box 56, Dunedin, 9054 New Zealand

**Keywords:** Reaction times, Statistical power, Within-subjects designs, Sample size, Number of trials, Practice effects

## Abstract

Due to limitations in the resources available for carrying out reaction time (RT) experiments, researchers often have to choose between testing relatively few participants with relatively many trials each or testing relatively many participants with relatively few trials each. To compare the experimental power that would be obtained under each of these options, I simulated virtual experiments using subsets of participants and trials from eight large real RT datasets examining 19 experimental effects. The simulations compared designs using the first $N_T$ trials from $N_P$ randomly selected participants, holding constant the total number of trials across all participants, $N_P \! \times \! N_T$. The $[N_P,N_T]$ combination maximizing the power to detect each effect depended on how the mean and variability of that effect changed with practice. For most effects, power was greater in designs having many participants with few trials each rather than the reverse, suggesting that researchers should usually try to recruit large numbers of participants for short experimental sessions. In some cases, power for a fixed total number of trials across all participants was maximized by having *as few as two* trials per participant in each condition. Where researchers can make plausible predictions about how their effects will change over the course of a session, they can use those predictions to increase their experimental power.

Researchers planning reaction time (RT) studies must often consider a trade-off between the number of participants ($N_P$) and the number of trials per participant in each condition ($N_T$). Naturally it is desirable to have as many of each as possible, but when resources are limited, researchers may be forced to choose between a large number of participants with few trials each, a small number of participants with many trials each, or medium numbers of both participants and trials. These different options might provide distinctly different levels of experimental power (Baker et al., 2021; Brysbaert & Stevens, 2018; Rouder & Haaf, 2018), so it seems worthwhile to compare the power of different options, especially in light of the importance of maximizing power for the reproducibility of scientific results (e.g., Button & Munafò, 2017).

For example, assume that a researcher wants to compare the mean RTs of two conditions with a paired *t*-test and has the resources to collect 500 trials per condition. Which would have greater statistical power: a study with 10 participants and 50 trials per condition (henceforth denoted as $[{10}_P,{50}_T]$), a study with 25 participants and 20 trials per condition ($[{25}_P,{20}_T]$), or a study with 50 participants and 10 trials per condition ($[{50}_P,{10}_T]$)?

The trade-off between the number of participants $N_P$ and the number of trials $N_T$ is particularly salient in the current research environment because of the increasing popularity of on-line experiments (e.g., Hilbig, 2016; Kochari, 2019; Ratcliff & Hendrickson, 2021; Semmelmann & Weigelt, 2017). Participants in these experiments are generally paid at a fixed hourly rate, so the total participant cost is determined by the total number of trials $N_P \! \times \! N_T$ regardless of how the trials are divided across participants. When trying to maximize statistical power at a fixed cost, the question of how to divide trials across participants is a very practical one.

The power to detect an effect on RT with a paired *t*-test is generally modelled as a function of three properties of the effect (e.g., Rouder & Haaf, 2018). The first is the effect’s true size, $\mu ^{}_{\Delta }$, which is theoretically the between-condition difference in mean RTs on average across infinite numbers of participants and trials. Other things being equal, power is larger for larger values of $\mu ^{}_{\Delta }$. The second property is the variability across participants in the effect’s true size, $\sigma ^{}_{\scriptscriptstyle P}$. The idea is that there is some true effect size $\Delta _p$ for each participant *p*, which could be measured by collecting an infinite number of trials from that participant. The parameter $\sigma ^{}_{\scriptscriptstyle P}$ is the standard deviation of these $\Delta _p$ values across an infinite number of participants. Power tends to be larger when there is less of this participant-to-participant variation (i.e., smaller $\sigma ^{}_{\scriptscriptstyle P}$), because more consistent effects are easier to detect. The third property is the trial-to-trial variability of an individual participant’s RTs within a condition, $\sigma ^{}_{\scriptscriptstyle T}$, which reflects the pure random noise in the single-trial RT measurements themselves. This variability could arise from momentary fluctuations in the participant’s state (i.e., cognitive, physiological, etc.), from trial-to-trial variations in stimulus presentation (e.g., stimulus positions in a visual search task), and from hardware timing inaccuracies (e.g., those associated with video display and keyboard scanning).

Using this basic three-component model, Rouder and Haaf (2018) showed that the power of paired *t*-tests is always greater when $N_P$ is larger, for any fixed total number of trials $N_P \! \times \! N_T$, but that the power advantage for larger $N_P$ is relatively small when the trial-to-trial RT variability $\sigma ^{}_{\scriptscriptstyle T}$ is much larger than the person-to-person variation in true effect size $\sigma ^{}_{\scriptscriptstyle P}$. In other words, to the extent that the effect is the same for all participants, it may be possible to show it with only a few participants provided that there are many trials from each participant. They argued that the trial-to-trial RT variability $\sigma ^{}_{\scriptscriptstyle T}$ would usually be much larger than person-to-person variation in true effect size $\sigma ^{}_{\scriptscriptstyle P}$, and they concluded that researchers could usually test fewer participants with more trials each—which is generally the more convenient option for in-lab experiments—without losing much power relative to designs with more participants tested for fewer trials each.

Although Rouder and Haaf’s (2018) conclusions from the standard model are suggestive, it is difficult to be certain how they would apply in any given planned experiment. A major pragmatic problem is that the sizes of $\sigma ^{}_{\scriptscriptstyle P}$ and $\sigma ^{}_{\scriptscriptstyle T}$ are generally unknown. Since the size of the power advantage for larger $N_P$ values depends on these quantities, it is difficult to estimate how much power would be sacrificed by using a larger $N_T$ instead. More importantly, the standard model does not allow for practice effects. It effectively assumes that none of the effect size and variability parameters (i.e., $\mu ^{}_{\Delta }$, $\sigma ^{}_{\scriptscriptstyle P}$, and $\sigma ^{}_{\scriptscriptstyle T}$) change with practice, which need not be the case. This assumption is important, because differences in $N_T$ necessarily entail differences in the amount of practice. Conclusions from the standard model must therefore be limited to paradigms for which this “no changes with practice” assumption is realistic.

In fact, the sizes of some effects have been shown to change as participants get more practice in a task (e.g., Klapp, 1995; Ruthruff et al., 2001; Shiffrin & Schneider, 1977; Worringham & Stelmach, 1990). This is not surprising, because some effects may develop only after sufficient training, and others may diminish as participants learn to cope better with the more difficult conditions. The variability parameters $\sigma ^{}_{\scriptscriptstyle P}$ and $\sigma ^{}_{\scriptscriptstyle T}$ can also change with practice. As Smith and Little (2018) put it, “Researchers who do small-N [i.e., small $N_P$] studies would agree that ... within-observer and between-observer variability [i.e., $\sigma ^{}_{\scriptscriptstyle T}$ and $\sigma ^{}_{\scriptscriptstyle P}$] both decrease progressively with increasing time on task” (p. 2,087). To the extent that there are changes with practice in the values of the underlying parameters $\mu ^{}_{\Delta }$, $\sigma ^{}_{\scriptscriptstyle P}$, and $\sigma ^{}_{\scriptscriptstyle T}$, these changes also need to be considered in modelling changes in power across [$N_P$,$N_T$] combinations.

As opposed to a mathematical analysis like that of Rouder and Haaf (2018), an alternative approach to the $N_P$ versus $N_T$ trade-off question is purely empirical: The question can be investigated by comparing directly the results of different real studies with many participants and few trials per participant, or the reverse. If empirical studies of these two types were compared, then there would be no need for the assumptions and simplifications required by the mathematical approach, because the observed RTs would by definition reflect realistic effect sizes, effect size variability, trial-to-trial RT variability, practice effects, and so on.

Of course it would be impractical to collect new data for many $[{10}_P,{50}_T]$ studies and many $[{50}_P,{10}_T]$ studies to see which $[N_P,N_T]$ combination had the higher power in practice. Fortunately, the equivalent comparison can be mimicked almost exactly with virtual experiments constructed by taking subsamples of participants and trials from published “mega-studies” having very large numbers of participants and trials per participant (e.g., Miguel-Abellaetal et al., 2022). For example, to compare the power of $[{10}_P,{50}_T]$ versus $[{50}_P,{10}_T]$ studies to detect a certain effect within a given dataset, one could look at the power of virtual studies with randomly sampled subsets of $N_P=10$ or $N_P=50$ participants. For each randomly sampled participant, only the first $N_T=50$ or the first $N_T=10$ trials per condition, respectively, would be included in the analysis to mimic the findings with more versus fewer trials per participant. Given that these are actual observed RTs, the only assumption required by this procedure is that the RTs in the first $N_T$ trials from a given participant do not depend on the number of additional trials that the participant will perform subsequently within the study.

Baker et al. (2021) used a similar approach of random sampling from an existing dataset to study the power of a *t*-test to detect an attentional cuing effect with different numbers of participants and trials. Unfortunately, this was a small dataset ($N_P=38$), so they had to sample trials randomly rather than taking the first $N_T$ trials from each participant, thus ignoring possible practice effects (e.g., changes in effect size or variability with changes in $N_T$). They did not explicitly compare scenarios with fixed total numbers of trials $N_P \! \times \! N_T$, but they concluded that adequate power to detect the large attentional effect could be obtained with approximately $N_P=20$ and $N_T=10$ or $N_P=8$ and $N_T=50$. As will be considered in the General Discussion, Brysbaert and Stevens (2018) also used a sampling approach to study the relation of power to the numbers of participants and trials in psycholinguistic studies analyzed with linear mixed effects (LME) models—a type of analysis that allows for the presence of two random factors (i.e., participants and items) and is more complex than the *t*-test analyses considered by Rouder and Haaf (2018) and addressed in the present article.

To obtain a broader picture of the $N_P$ versus $N_T$ trade-off, the present simulations examined the power to detect 19 experimental effects within eight different datasets. For each effect, power was examined across varying $[N_P,N_T]$ combinations with a constant total number of trials $N_P \! \times \! N_T$ to see which combinations produced the highest power to detect that effect with the given total number of trials. Only the first $N_T$ trials from each participant in each condition were used to control for practice effects, and the results showed that power differences among the different $[N_P,N_T]$ combinations depend critically on how the experimental effect under study changes with practice. In brief, the simulations showed that for most effects (14/19) power was better with large $N_P$ and small $N_T$ than with the reverse. In addition, power was approximately the same with large $N_P$ and small $N_T$ as with the reverse for four of the effects, and it was better with small $N_P$ and large $N_T$ than the reverse for only one of the effects. Thus, to the extent that these available datasets are representative of RT research in general, the present results suggest that researchers can most often increase power by opting for large $N_P$ and small $N_T$.

## Megastudy of Hutchison et al. (2013)

The Semantic Priming Project (SPP) dataset of Hutchison et al. (2013) has lexical decision task RTs for visually presented letter strings with more than 500 participants and more than 1,500 trials per participant. One effect in their data was that responses were substantially faster to words than to nonwords, and simulations can reveal how often this effect would be significant using only the first $N_T$ trials from randomly selected subsets of $N_P$ participants with various $[N_P,N_T]$ combinations. For these simulations, it is important to choose $[N_P,N_T]$ combinations and $\alpha$ cutoffs that result in power values across much of the possible 0–1 range. If power values were all at or very close to the ceiling of 1.0, for example, it would be difficult to see any power differences between the different $[N_P,N_T]$ combinations.

After some trial and error to choose an $[N_P,N_T]$ combination and $\alpha$ level that would yield only intermediate power levels with this strong word/nonword effect, for an initial test I simulated 100,000 virtual studies with a random subset of $N_P=10$ participants, including only the first $N_T=20$ trials from each participant in each condition (i.e., words versus nonwords) and checking for a statistically significant effect at $\alpha =0.0001$ (two-tailed). In this simulation, 11.5% of the virtual experiments yielded a significant RT difference between words and nonwords (i.e., the known real condition effect was correctly detected). For comparison, I then simulated 100,000 virtual studies with $N_P=20$ using the first $N_T=10$ trials in each condition for each randomly selected participant, and 57.8% of these produced significant effects with the same $\alpha$. Thus, the results of these virtual experiments indicate that—at least under conditions comparable to those of the SPP study—researchers would have much more power to detect a word/nonword effect on mean RT with $N_P=20$ and $N_T=10$ than with the reverse.

**Fig. 1:**
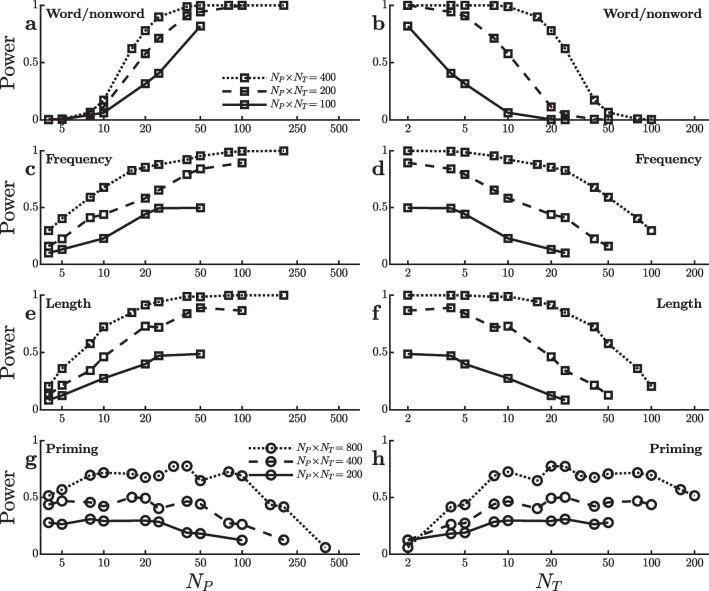
Power to detect an effect on mean reaction time in virtual studies with different numbers of participants ($N_P$) and trials per condition ($N_T$) in the dataset of Hutchison et al. (2013). Each line reflects virtual studies with the same total number of trials per condition (i.e., $N_P \! \times \! N_T$), with square and circle symbols used to indicate different total numbers. **a** and **b** Power to detect a word/nonword effect. **c** and **d** Power to detect a word frequency effect. **e** and **f** Power to detect a word length effect. **g** and **h** Power to detect a semantic priming effect

Figure 1a and b trace out analogous power curves using $\alpha =0.0001$ with a range of [$N_P$,$N_T$] combinations producing $N_P \! \times \! N_T = 200$ trials per condition, and also analogous curves with combinations yielding $N_P \! \times \! N_T = 100$ or 400. The curves showing power as a function of $N_T$ are essentially left-to-right reversals of those showing power as a function of $N_P$, because $N_P$ and $N_T$ are inversely related to each other when the total $N_P \! \times \! N_T$ is held constant. Despite that, the figures are not mirror-images of one another because the horizontal axes have different ranges. Analogous curves depicting the results with two power-related measures that are independent of the $\alpha$ level (i.e., confidence interval width and average *Z*-score of the attained *p* level) are shown in the appendix.

Figure 1a shows that the power to detect the word/nonword effect increases steadily with $N_P$ for all three total trial numbers. Viewing the same power levels in terms of their relations to the complementary $N_T$ values (Fig. 1b), power seems maximal with smaller numbers of trials per participant in each condition—because there are correspondingly more participants—remarkably all the way down to two trials. Thus, the earlier $[{10}_P,{20}_T]$ versus $[{20}_P,{10}_T]$ comparison generalizes across a range of $[N_P,N_T]$ combinations.

The results shown in Fig. 1a and b may be specific to the word/nonword effect within the SPP dataset. It is important to ask whether similar relationships of power to $N_P$ and $N_T$ are also found in other situations. Therefore, simulations comparable to those testing for the word/nonword effect were also run to test for two other effects present in the SPP data—word length and word frequency effects. These effects were smaller than the word/nonword effect, so these used the more lenient $\alpha =0.01$ to keep power in an intermediate range. Adjustment of the $\alpha$ level effectively counteracts changes in numerical effect size so that power levels are comparable for testing a numerically larger effect at a smaller $\alpha$ and testing a numerically smaller effect at a larger $\alpha$. The results of these simulations indicate that power also tends to increase with $N_P$ when testing for the word length and word frequency effects (Fig. 1c–f). Thus, when testing for word/nonword, word length, or word frequency effects, power is maximized by spreading the trials over as many participants as possible, with no sign that power starts to decrease when the number of trials per participant is too small.

Finally, there was also a highly significant effect of semantic priming in the SPP dataset, and further simulations were carried out to examine the power to detect this effect with various $[N_P,N_T]$ combinations. This effect was numerically much smaller than the other effects, so these simulations used $\alpha =0.05$ and larger total numbers of trials $N_P \! \times \! N_T$, thus again adjusting the simulation conditions rather than the RTs to produce intermediate power levels so that power differences among the combinations would not be obscured by floor or ceiling effects. Interestingly, the relation of power to $[N_P,N_T]$ combinations is different for the semantic priming effect, as shown in Fig. 1g and h. For this effect, power is fairly stable or increases only slightly as $N_P$ increases up to approximately $N_P=50$, and then power decreases. Viewing the same power levels in terms of their relations to the complementary $N_T$ values (Fig. 1h), power seems maximal at approximately 20 trials per participant in each condition, with little decrease in power if $N_T$ is increased beyond that point (despite corresponding decreases in $N_P$).

**Fig. 2:**
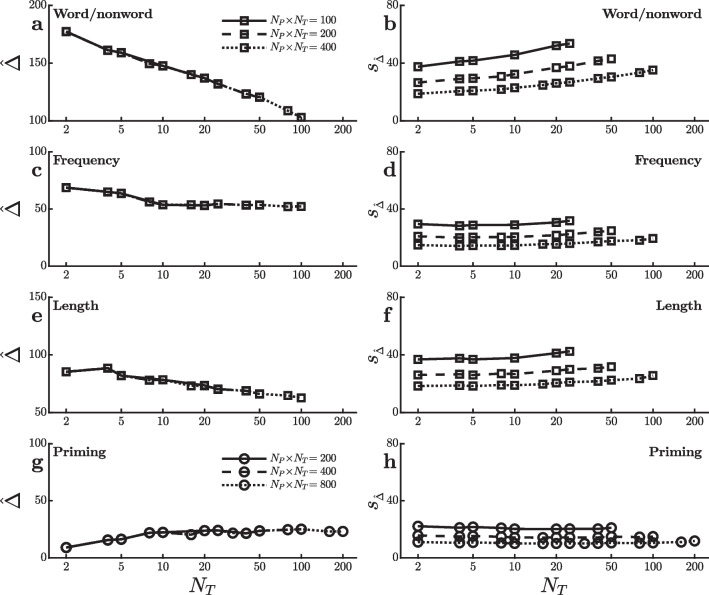
Mean ($\hat{\Delta }$) and standard error ($\sigma ^{}_{\scriptscriptstyle \hat{\Delta }}$) of the reaction time effect size in ms for the virtual studies shown in Fig. 1

To understand what causes the difference in power trends for the semantic priming effect versus the other effects, it is helpful to look separately at the two quantities which determine the value of the *t*-test used in H0 testing (i.e., $t = \hat{\Delta } / s^{}_{\scriptscriptstyle \hat{\Delta }}$). The *t* value is larger—and H0 is thus more likely to be rejected—when the observed mean effect size, $\hat{\Delta }$, is larger and when the estimated standard error of this effect size, $s^{}_{\scriptscriptstyle \hat{\Delta }}$, is smaller. Thus, power is affected by changes in either of these quantities with the amount of practice (i.e., $N_T$). Figure 2 shows how each of them actually changed with $N_T$ within the SPP dataset for the word/nonword, word length, word frequency, and semantic priming effects.

Figure 2a, c, e, and g show the mean effect sizes, $\hat{\Delta }$ (in ms), for each of the different effects as a function of the number trials used to assess the effect. For example, the $\hat{\Delta }$ values with $N_T=30$ and $N_T=50$ indicate the mean effect sizes computed across all of the virtual experiments using just the first 30 or just the first 50 trials in each condition. Note that these means do not vary with the total number of participants (except for the variation associated with the random sampling of participants) because the averages across 100,000 samples tend to be close to the population average regardless of sample size. This is why the lines for the different total numbers of trials $N_P \! \times \! N_T$ are essentially superimposed. The mean effect sizes shown in Fig. 2a, c, e, and g reveal differences in how these four effects change with practice. For example, the word/nonword effect is by far the largest early in practice, with the effect reduced to only approximately half of its original magnitude after 100 trials. Thus, the power to detect a word/nonword effect would tend to be especially large when $N_T$ is small, because that is when the effect itself is numerically the largest. The same is true, albeit to a lesser extent, for the word frequency and word length effects. In contrast, the priming effect shown in Fig. 2g *increases* with practice. This effect is barely above zero when measured with only two trials per condition for each participant, reaching its full size only when there are at least 10–20 trials. For this effect, then, power will tend to be low with small $N_T$ and correspondingly large $N_P$ simply because $[N_P,N_T]$ combinations with small $N_T$ test for the effect when it is numerically small—exactly the opposite of the word/nonword, word frequency, and word length effects^1^. In sum, the differential changes across practice in the sizes of the word/nonword, word frequency, word length, and semantic priming effects shown in Fig. 2a, c, e, and g can explain at least part of the difference between these effects in how power changes across different $[N_P,N_T]$ combinations.

Figure 2b, d, f, and h show how the standard error of each effect, $s^{}_{\scriptscriptstyle \hat{\Delta }}$, is related to practice (i.e., $N_T$)^2^. The effect of $N_P$ can be seen in these plots as the difference between the lines at each $N_T$ value, illustrating the fact that—other things being equal—the standard error of a mean difference decreases with increases in the number of participants.

Looking first at the word/nonword effect, Fig. 2b shows a clear tendency for this effect’s standard error to increase with the number of trials $N_T$ (i.e., with decreasing $N_P$ within a fixed $N_P \! \times \! N_T$). Since power decreases as standard error increases, this increase in standard error with practice affects power in the same way as the decreasing mean effect size with practice (Fig. 2a)—that is, it also tends to make power smaller with smaller $N_P$ and larger $N_T$. The same is true for the word frequency and word length effects (Fig. 2d and f), although to a lesser extent. In contrast, the standard error of the semantic priming effect does not increase with $N_T$ and may even decrease slightly (Fig. 2h), so the power to detect this effect would not be reduced by inflation of the standard error at the larger $N_T$ values as seen with the other effects. In sum, the differential changes across practice in the variabilities of the word/nonword, word frequency, word length, and semantic priming effects also appear to contribute to the difference between effects in their relations of power to $[N_P,N_T]$ combinations.

Why does the standard error of the effect size increase with $N_T$ for the word/nonword, word length, and word frequency effects but not the semantic priming effect? In theory, the standard error of an effect is

1 $$\begin{aligned} \sigma ^{}_{\scriptscriptstyle \hat{\Delta }} = \sqrt{ \frac{\sigma ^2_{\scriptscriptstyle P}}{N_P} + \frac{2\sigma ^2_{\scriptscriptstyle T}}{N_P \cdot N_T} } \end{aligned}$$

(e.g., Baker et al., 2021; Rouder & Haaf, 2018)^3^. This value increases with the amount of participant-to-participant variability in the individual participants’ true effects, $\sigma ^{}_{\scriptscriptstyle P}$, and with the amount of trial-to-trial variability in each individual’s RTs within a condition, $\sigma ^{}_{\scriptscriptstyle T}$. It decreases with increases in both $N_P$ and $N_T$. As Rouder and Haaf (2018) emphasized, the influence of $N_P$ is stronger than that of $N_T$, because both variances are divided by $N_P$ but only $\sigma ^2_{\scriptscriptstyle T}$ is divided by $N_T$. If $\sigma ^{}_{\scriptscriptstyle P}$ is small relative to $\sigma ^{}_{\scriptscriptstyle T}$, though, this difference between $N_P$ and $N_T$ is not very important. In the limit of $\sigma ^{}_{\scriptscriptstyle P}=0$, the standard error decreases with the product $N_P \! \times \! N_T$ regardless of how this product is formed by a particular [$N_P$,$N_T$] combination. Thus, the relatively flat lines in Fig. 2h suggest that $\sigma ^{}_{\scriptscriptstyle P}$ is small relative to $\sigma ^{}_{\scriptscriptstyle T}$ for the semantic priming effect—that is, the effect is about the same size for all participants—whereas the increases seen in Fig. 2b, d, and f imply that $\sigma ^{}_{\scriptscriptstyle P}$ is larger than $\sigma ^{}_{\scriptscriptstyle T}$ for the other three effects.

Overall, the results shown in Figs. 1 and 2 suggest a preliminary generalization about the best way to divide a fixed total number of trials $N_P \! \times \! N_T$ across participants versus trials when testing for a condition effect on mean RT. It appears that larger numbers of participants are generally preferable, especially when the number of trials per condition is at least $N_T=20$ or so. Larger numbers of participants seem especially important when the effect gets smaller or more variable with practice. To investigate the generality of this conclusion further, I conducted analogous virtual experiments using the data from several other large studies with additional tasks and condition effects.

**Fig. 3:**
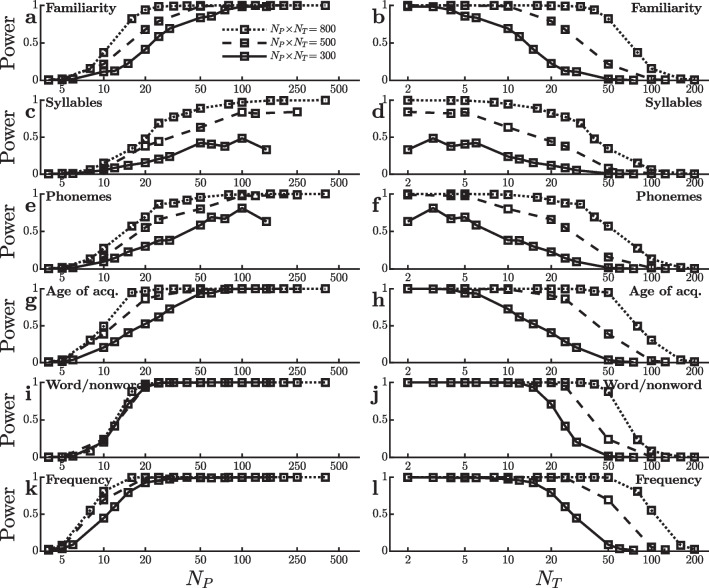
Power to detect an effect on mean reaction time in virtual studies with different numbers of participants ($N_P$) and trials per condition ($N_T$) in the dataset of Goh et al. (2020). Each line reflects virtual studies with the same total number of trials per condition (i.e., $N_P \! \times \! N_T$). **a** and **b** Power to detect a word familiarity effect. **c** and **d** Power to detect an effect of the number of syllables. **e** and **f** Power to detect an effect of the number of phonemes. **g** and **h** Power to detect an effect of age of acquisition (acq.). **i** and **j** Power to detect a word/nonword effect. **k** and **l** Power to detect a word frequency effect

## Megastudy of Goh et al. (2020)

The lexical decision task megastudy of Goh et al. (2020) provides another rich dataset for examining the trade-off between the numbers of participants and trials. These researchers collected approximately 4,000 RTs from each of more than 400 participants. The study differed from that of Hutchison et al. (2013) in that it used auditory rather than visual stimulus presentation. The results showed large (50–100 ms) effects of word familiarity, number of syllables, number of phonemes, age of acquisition, word/nonword status, and word frequency. I simulated virtual experiments examining the power to detect each of these effects with different $[N_P,N_T]$ combinations, using $\alpha =0.0001$ to avoid the power ceiling because these effects were all large.

Figure 3 shows the results of these virtual experiments, and their consistency across effects is striking. For each effect, power increases essentially monotonically with $N_P$, showing massive power gains from approximately $N_P=10$ to $N_P=30$ in nearly all cases. Thus, the virtual experiments conducted using the data from Goh et al. (2020) reinforce the preliminary suggestion that power tends to be optimized by dividing a fixed total number of trials $N_P \! \times \! N_T$ across a large number of participants even if that means only a small number of trials per participant can be collected due to resource limitations.

There was one interesting anomaly in the virtual experiments conducted with the dataset of Goh et al. (2020). Surprisingly, Fig. 3i shows that the power curves for detecting the word/nonword effect are virtually superimposed for 300, 500, or 800 total trials per condition. With $N_P=10$ participants, for example, power does not seem to depend on whether there are 30, 50, or 80 trials per condition for each participant. How is this possible, given that averaging more trials necessarily produces statistically more stable results?

**Fig. 4:**
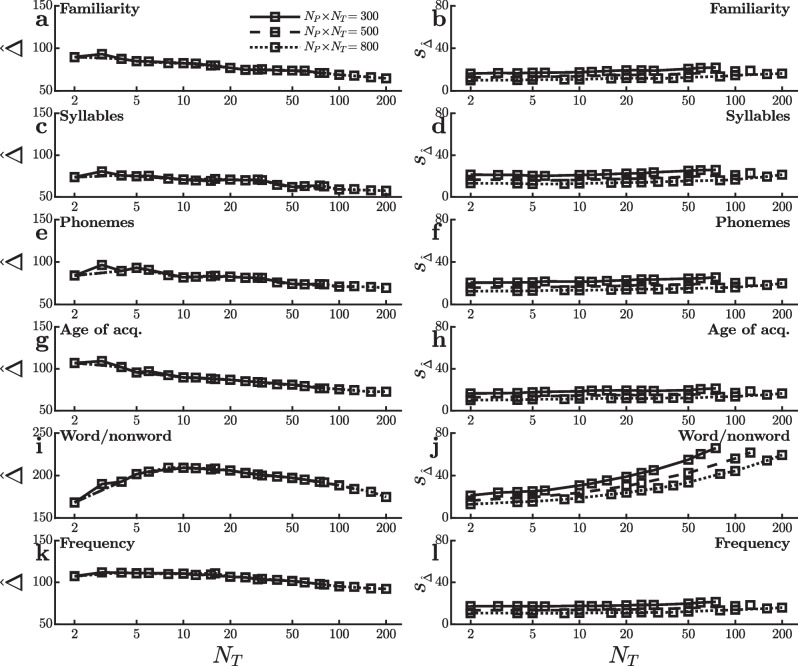
Mean ($\hat{\Delta }$) and standard error ($\sigma ^{}_{\scriptscriptstyle \hat{\Delta }}$) of the reaction time effect size in ms for the virtual studies shown in Fig. 3

To understand the causes of this anomaly, it is useful to again look separately at the two quantities $\hat{\Delta }$ and $s^{}_{\scriptscriptstyle \hat{\Delta }}$ that determine the value of the *t*-tests. Figure 4 shows how each of these two measures changes as a function of practice (i.e., $N_T$). Critically, Fig. 4i shows that the word/nonword effect is noticeably larger when it is measured using the first 30 trials, somewhat smaller when it is measured using the first 50 trials, and smaller still when it is measured using the first 80 trials. This is the same decrease in word/nonword effect size seen with the SPP dataset (Fig. 2a), and it would again work against the power increase that would normally be expected as $N_T$ increases from 30 to 80 with a constant $N_P=10$. This same argument explains the lack of $N_T$ effect throughout the $N_P$ range of Fig. 4i where power values are intermediate between floor and ceiling (i.e., approximately $N_P=10$–20), because these $N_P$ values correspond to $N_T=15$–80 trials and the word/nonword effect size decreases throughout this $N_T$ range (Fig. 4i). Although the effect size also decreases at the lowest levels of practice ($N_T=2$–10), this decrease is not visible in the power values of Fig. 3i because there are so many participants with these $N_P \! \times \! N_T$ values (i.e., $N_P \ge 30$) that power is at ceiling. As is evident in Fig. 4a, c, e, g, and k, however, other effects also decrease with practice over this range, albeit less so. Thus, the especially large decrease in word/nonword effect size with practice may only partially explain the anomaly.

The curves in the panels on the right side of Fig. 4 show the estimated standard errors of the effect sizes, $s^{}_{\scriptscriptstyle \hat{\Delta }}$. As was the case in the SPP dataset, the standard error of the word/nonword effect size (Fig. 4j) increases with the number of trials used to measure it. Since power decreases as standard error increases, this trend works against the power increase expected with more trials and thus also contributes to the anomaly seen in Fig. 3i. In contrast, the standard errors of the other effects vary little with the number of trials (Fig. 4b, d, f, h, and l). As was discussed earlier in connection with Eq. 1 and the SPP dataset, the different patterns of $s^{}_{\scriptscriptstyle \hat{\Delta }}$ versus $N_T$ in Fig. 4 suggest that the size of the word/nonword effect varies somewhat across participants (i.e., large $\sigma ^{}_{\scriptscriptstyle \hat{\Delta }}$) but that the sizes of the other effects are fairly stable across participants (i.e., small $\sigma ^{}_{\scriptscriptstyle \hat{\Delta }}$).

**Fig. 5:**
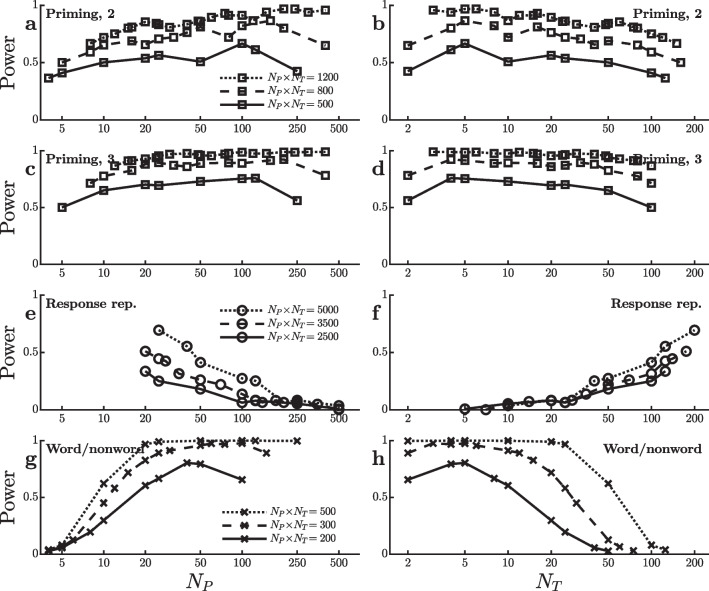
Power to detect an effect on mean reaction time in virtual studies with different numbers of participants ($N_P$) and trials per condition ($N_T$) in the dataset of Adelman et al. (2014). Each line reflects virtual studies with the same total number of trials per condition (i.e., $N_P \! \times \! N_T$). **a** and **b** Power to detect an orthographic priming effect based on the two-category distinction of Brysbaert and Stevens (2018). **c** and **d** Power to detect an orthographic priming effect based on the three-category distinction of Brysbaert and Stevens (2018). **e** and **f** Power to detect an effect of response repetition (rep.). **g** and **h** Power to detect a word/nonword effect

## Megastudy of Adelman et al. (2014)

The present analyses were also applied to data from the orthographic priming study of Adelman et al. (2014), which had also been used in the LME-based simulations of Brysbaert and Stevens (2018). In each trial of this study, participants saw a prime stimulus of lower-case letters followed by a target stimulus of upper-case letters, and they were required to give a lexical decision response to the target. Adelman et al. (2014) compared 28 different prime types based on the patterns of matching versus mismatching letter positions of the prime and target, and they obtained approximately 800 RTs from each of approximately 1,000 participants. Following Brysbaert and Stevens (2018), I looked at a two-condition priming effect with word targets comparing the fastest 14 versus the slowest 14 prime types, and a priming effect based on three conditions which compared the fastest and slowest prime types while excluding prime types with intermediate mean RTs^4^. Both priming effects were small, so the virtual experiments used reasonably large numbers of trials and $\alpha =0.05$. As shown in Fig. 5a–d, the power to detect these effects was not much affected by the $[N_P,N_T]$ combination, just as Brysbaert and Stevens (2018) found with the LME analysis, although of course it was affected by the total number of trials $N_P \! \times \! N_T$.

**Fig. 6:**
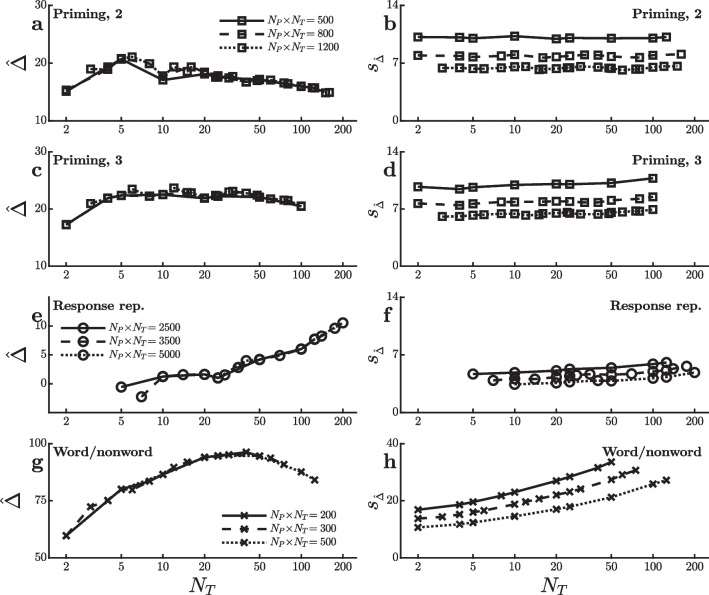
Mean ($\hat{\Delta }$) and standard error ($\sigma ^{}_{\scriptscriptstyle \hat{\Delta }}$) of the reaction time effect size in ms for the virtual studies shown in Fig. 5

Two further experimental effects can be seen in the full dataset of Adelman et al. (2014)^5^. One is a very small response repetition effect, with faster RTs when a response is the same as that given in the previous trial than when it is different. The other is a very large word versus nonword effect, with faster responses to words. Fig. 5e–h show how the power to detect these effects (with $\alpha =0.05$ and $\alpha =0.0001$, respectively) depend on the $[N_P,N_T]$ combination. In contrast to the priming effects in this dataset, power to detect the response repetition and word/nonword effects both differ substantially across $[N_P,N_T]$ combinations, but in opposite directions. To find the response repetition effect, power is better with a smaller number of participants tested extensively (at least 100 trials in both the repetition and nonrepetition conditions). When looking for the word/nonword effect, in contrast, it seems that only about five trials per participant are needed in each condition, with increases in the number of participants being much more helpful for increasing power.

Figure 6 shows how $\hat{\Delta }$ and $s^{}_{\scriptscriptstyle \hat{\Delta }}$ change as a function of practice (i.e., $N_T$) for all four of the effects, again providing clues as to the reasons for the different patterns of power in Fig. 5. As shown in Fig. 6a–d, both the means and standard errors of the priming effects are rather constant across $N_T$, consistent with the small changes in power across $[N_P,N_T]$ combinations. In contrast, the mean response repetition effect (Fig. 6e) depends strongly on $N_T$. This effect is virtually absent unless participants are tested with at least 100 trials per condition, so there is little power to detect it with large $N_P$ and small $N_T$. The word/nonword effect also grows over the first 30–40 trials (Fig. 6g), but it is nonetheless large enough in the initial trials to be detected with small $N_T$, partly because its standard error is smallest in that case (Fig. 6h).

**Fig. 7:**
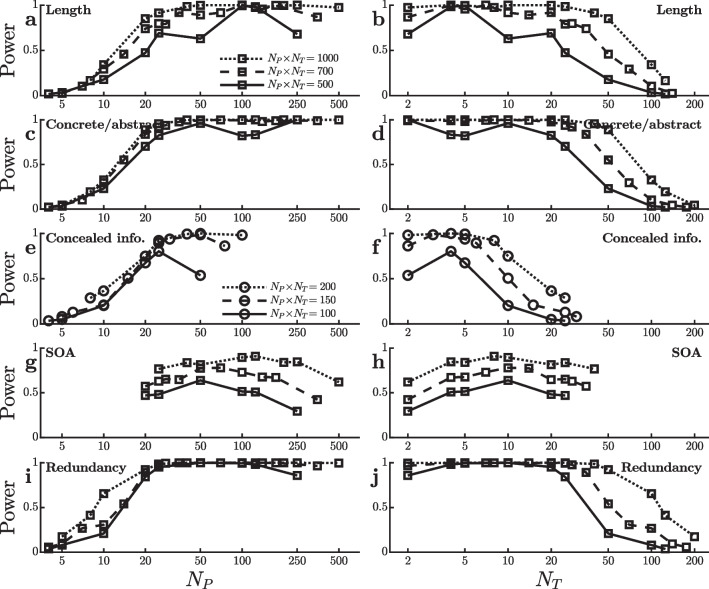
Power to detect an effect on mean reaction time in virtual studies with different numbers of participants ($N_P$) and trials per condition ($N_T$). Each line reflects virtual studies with the same total number of trials per condition (i.e., $N_P \! \times \! N_T$), with square and circle symbols used to indicate different total numbers. **a** and **b** Power to detect a word length effect in the dataset of Miguel-Abella et al. (2022). **c** and **d** Power to detect a difference between concrete and abstract words in the dataset of Pexman et al. (2017). **e** and **f** Power to detect a concealed information (info.) effect in the dataset of Lubczyk et al. (2022). **g** and **h** Power to detect an effect of stimulus onset asynchrony (SOA) in the dataset of Bazilinskyy and De Winter (2018). **i** and **j** Power to detect an effect of redundancy in the dataset of Wales (2014)

**Fig. 8:**
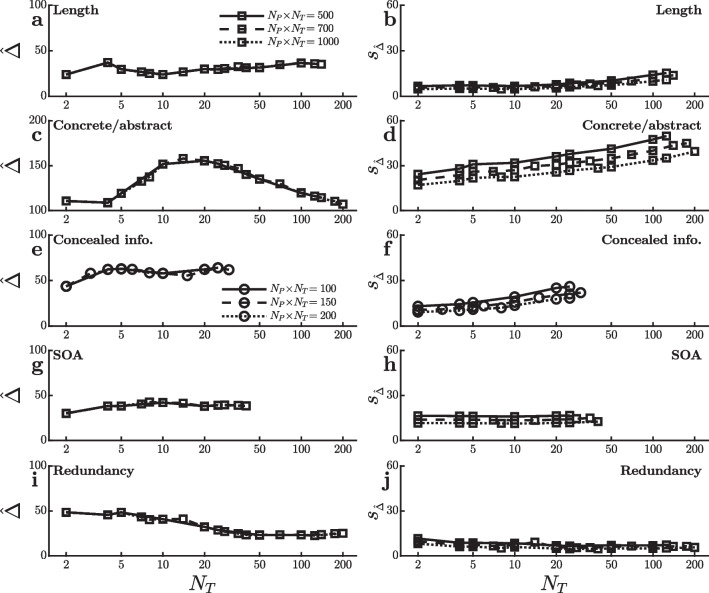
Mean ($\hat{\Delta }$) and standard error ($\sigma ^{}_{\scriptscriptstyle \hat{\Delta }}$) of the reaction time effect size in ms for the virtual studies shown in Fig. 7

## Additional Datasets

Figure 7 shows power curves obtained in virtual experiments with additional datasets chosen to expand the range of tasks being examined as widely as possible, and Fig. 8 shows the corresponding mean effect sizes and standard errors. Figure 7a and b used data from the Spanish verb reading megastudy reported by Miguel-Abella et al. (2022). The participants’ task was simply to read aloud a visually presented verb as quickly as possible, and RT was measured from the onset of the word to the onset of the vocalization. Responses were approximately 35 ms faster to shorter words than to longer ones (i.e., 1–7 versus 9+ characters), and the present simulations looked at the power of virtual studies to detect this word length effect with $\alpha =0.001$ and total numbers of trials adjusted to avoid ceiling effects on power. Power generally increases with the number of participants, although it then decreases slightly when the number of trials per condition dips below five, despite the large $N_P$ values in these cases (i.e., $N_P\approx 100$–500 for the three $N_P \! \times \! N_T$ values in the graph). As with the similar dip seen with the semantic priming effect (Fig. 1g and h), this could be because the length effect is slightly smaller very early in practice (Fig. 8a).

Figure 7c and d used data from the megastudy of Pexman et al. (2017). The stimuli were single visually-presented words, and participants made speeded judgments of whether each word referred to something concrete versus abstract, with average RT approximately 80 ms less for concrete words than for abstract ones. Simulations assessed the power to detect this concrete/abstract effect with various [$N_P$,$N_T$] combinations and $\alpha =0.001$, and power again increased with the number of participants, despite the fact that the effect was smallest—though still large in absolute terms—in the initial trials (Fig. 8c). To some extent this may have been due to the fact that the standard error of the effect was smallest in the initial trials (Fig. 8d). In any case, the high levels of power with very small $N_T$ values seen in this simulation may be somewhat deceptive on procedural grounds. Participants in this study were given 24 practice trials before the start of data collection, so the $N_T$ RTs shown on the abscissa of Fig. 7d did not come from the very first $N_T$ trials per condition—only the first $N_T$ trials recorded after this initial practice.

Figure 7e and f are based on data from the concealed information test (CIT) study of Lubczyk et al. (2022). For each participant, an item (i.e., a surname or date) that the participant regarded as familiar was selected as the target item. For the CIT test, participants were asked to make one response to that item and to some filler words referring to familiar or self-related concepts (e.g., “MINE”) but to make an alternative response to nontarget items including words relating to unfamiliar and other-related concepts (e.g., “OTHER”)^6^. The key CIT comparison was between “irrelevant” nontarget items selected to be unfamiliar to the participant versus a special “probe” nontarget item selected to be familiar (i.e., the participant’s own surname or birth date). Responses to the probe nontarget were approximately 75 ms slower than responses to the irrelevant nontargets, presumably because the probe’s specific familiarity to the participant interfered with its categorization with other unfamiliar items. Figure 7e and f show the power to detect this probe–irrelevant difference with various [$N_P$,$N_T$] combinations ($\alpha =0.001$). Power seems optimal with approximately five trials per participant in each condition after the initial training phase. This seems to be the number of trials at which the effect reaches its maximum size (Fig. 8e), and the standard error of the effect increases for larger $N_T$ values (Fig. 8f).

Figure 7g and h illustrate power curves obtained in virtual experiments using a dataset of simple RTs collected by Bazilinskyy and De Winter (2018). Participants were required to react as quickly as possible to the onset of any visual or auditory stimulus, and the main manipulation of interest was the stimulus onset asynchrony (SOA) between redundant stimuli presented on both modalities. On average, responses were 53 ms faster to redundant stimuli with short SOAs (SOA<100) as compared with long ones (SOA>100), and the present virtual experiments examined the power to detect this effect ($\alpha =0.01$). Due to the limited number of trials per participant, it was not possible to produce the indicated total trial numbers with the smaller $N_P$ values used with other datasets. The remarkable result with this dataset is that for a fixed $N_P \! \times \! N_T$ power depends very little on the [$N_P$,$N_T$] combination relative to the power fluctuations seen with other datasets. Power seems maximal with approximately 5–20 trials per participant in each condition and correspondingly approximately 20–100 participants, but the advantage for combinations in this range is quite small. Based on this effect’s small fluctuations across $N_T$ in effect size and standard error (Fig. 8g and h), it seems likely that the power to detect this effect is so stable across [$N_P$,$N_T$] combinations because the effect is quite consistent across both practice levels and participants.

Finally, Fig. 7i and j used data from a large, unpublished study conducted in my own lab (Wales , 2014). Participants in this study made simple RT responses to the onset of any visual stimulus, and stimuli were bright squares that could appear on the left of fixation, on the right of fixation, or redundantly on both sides. Responses were approximately 20 ms faster to redundant than single stimuli, and the simulations assessed the power to detect this redundancy gain ($\alpha =0.001$). For detecting the redundancy effect in this simple task, power again increased dramatically with the number of participants over the range of approximately 10–25. This can be attributed partly to the effect’s decrease with increasing practice (Fig. 8i).

## General Discussion

Across several large datasets with different RT tasks and experimental effects, the results of these virtual experiments indicate that in the majority of these cases experimental power to detect differences in mean RT with paired *t*-tests was greater with a relatively large number of participants, $N_P$, and a relatively small number of trials per participant in each condition, $N_T$, as compared with the reverse combination of a small $N_P$ and a large $N_T$. Of the 19 effects examined in Figs. 1, 3, 5, and 7, power increased strongly with $N_P$ in 14 cases, and power was not strongly affected by $N_P$ in four cases (priming effects in Figs. 1g, 5a, c and SOA effect in Fig. 7g). There was only one case in which power was clearly better with a small-$N_P$, large-$N_T$ combination (i.e., the response repetition effect in Fig. 5e). Judging from the current effects, then, the odds of increasing rather than decreasing power by using large $N_P$ rather than large $N_T$ appear to be approximately 14:1. These results thus suggest that—in the absence of indications to the contrary from previous research with similar paradigms—researchers wanting to maximize power should tend to place a greater emphasis on maximizing the number of participants rather than on getting a large number of trials per participant. For most of the real effects examined here, it appeared that 5–10 trials per participant in each condition were sufficient to obtain or closely approach maximum power for a given total number of trials $N_P \! \times \! N_T$, so it was advantageous to increase the number of participants rather than the number of trials per participant beyond this point. A natural corollary to this conclusion is that it will often be most efficient to provide participants with only a small number of warm-up or practice trials before starting data collection for tasks that are easy to learn.

A limitation of the present virtual experiments is that they used data from a relatively restricted subset of the many extant RT paradigms and effects. The data came primarily from psycholinguistic studies, where especially large datasets are most common. It is uncertain how widely the conclusions based on these studies can be generalized, because the $[N_P,N_T]$ trade-off could be different with other paradigms and effects. In fact, the diversity of $[N_P,N_T]$ trade-offs found even across the limited set of different effects examined here makes it clear that no simple recommendation for choosing an $[N_P,N_T]$ combination will optimize power for detecting all effects in all paradigms. But the tendency for power to increase somewhat consistently with $N_P$ across this subset of examples suggests that designs with a large $N_P$ would generally be a good place to start.

**Fig. 9:**
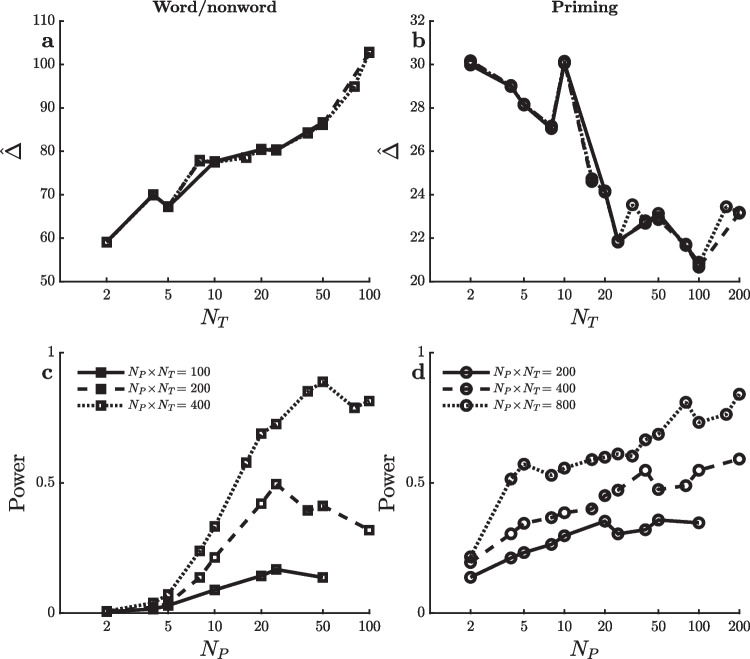
Results of simulations with modified versions of the dataset of Hutchison et al. (2013). **a** Mean size ($\hat{\Delta }$) of the word/nonword effect after reversing the order of the first 100 trials in each condition. **b** Mean size ($\hat{\Delta }$) of the semantic priming effect after reversing the order of the first 200 trials. **c** Power to detect the word/nonword effect after reversing the order of the first 100 trials. **d** Power to detect the semantic priming effect after reversing the order of the first 200 trials. Each line reflects virtual studies with the same total number of trials per condition (i.e., $N_P \! \times \! N_T$), with square and circle symbols used to indicate different total numbers

Even within the limited subset of paradigms considered here, there were striking exceptions to the general pattern of power increasing with $N_P$. Specifically, the present analyses show that the optimal $[N_P,N_T]$ combination depends on changes in effect size over the course of practice with the task. Figures 2, 4, 6, and 8 show that some of the effects studied here tended to increase with practice (i.e., with increasing $N_T$), some decreased, and some first increased and then decreased, with most of these practice effects being statistically reliable^7^. Such practice-related changes in effect size have substantial effects on the optimal $[N_P,N_T]$ combination. In particular, for some of the experimental effects examined here, part of the power advantage for $[N_P,N_T]$ combinations with relatively small $N_T$ values arose because the effects tended to be largest early in practice. In those cases, averaging across larger numbers of trials per participant actually *decreased* the size of the effect under study, which tended to reduce power. Such patterns highlight the importance of considering possible practice-related changes in effect size when estimating the power of an experimental design with any planned $[N_P,N_T]$ combination. Similarly, the patterns show that practice effects cannot safely be neglected in simulations comparing the power of different $[N_P,N_T]$ combinations.

To further illuminate the importance of practice effects, additional illustrative simulations were carried out with modified versions of the word/nonword and semantic priming datasets shown in Fig. 1a and g. These two datasets were chosen for these simulations because they show opposite effects of practice: the word/nonword effect decreased with practice (Fig. 2a) and the semantic priming effect increased with practice (Fig. 2g). The modified versions of both datasets were created by reversing the order of trials so that the practice effects would be reversed. Specifically, for the word/nonword effect, the modification was to reverse the order of the first 100 trials for each participant in each condition (i.e., the new trial 1 in each condition was the original trial 100 in that condition, the new trial 2 was the original trial 99, etc.). For the semantic priming effect, the first 200 trials per condition were reversed. Simulations parallel to those shown in Fig. 1 were then carried out with these modified datasets, and the results are shown in Fig. 9. As expected, once the trial orders are reversed, the word/nonword effect increases with practice (Fig. 9a) and the semantic priming effect decreases (Fig. 9b). More crucially, the relation of power to the $[N_P,N_T]$ combination changes markedly when the practice effects are reversed, as can be seen by comparing Fig. 9c and d with the corresponding Fig. 1a and g. Once the word/nonword effect increases with practice after reversal, the power to detect this effect no longer increases dramatically with larger numbers of participants as it did with the original dataset. Conversely, once the semantic priming effect decreases with practice after reversal, the power to detect it increases steadily with the number of participants, contrary to what was found with the original dataset. Thus, these simulations reinforce the point that it is especially important to increase $N_P$ relative to $N_T$ when the effect under study decreases with practice but not when it increases with practice.

The present conclusion that it is often especially important to increase $N_P$ rather than $N_T$ differs from that of Rouder and Haaf (2018), who argued that little power would be lost using $[N_P,N_T]$ combinations with smaller $N_P$ values under typical experimental conditions (see also, Smith & Little, 2018). For many of the real effects considered here, however, power was dramatically lower with $N_P=10$ than with $N_P=20$ or $N_P=50$, holding constant the total number of trials $N_P \! \times \! N_T$. It is not completely clear which discrepancies between Rouder and Haaf’s assumptions and the present real data are responsible for the differing conclusions about the power of different $[N_P,N_T]$ combinations, but the changes with practice shown in Figs. 2, 4, 6, and 8 provide important clues. First, most of the present effects changed with practice, though such practice effects were not included in Rouder and Haaf’s analysis. Second, for some effects the standard error of the effect size, $s^{}_{\scriptscriptstyle \hat{\Delta }}$, clearly increased with larger values of $N_T$. This is to be expected when the true effect size variability across participants, $\sigma ^{}_{\scriptscriptstyle P}$, is large relative to the variability of RT within a participant and condition, $\sigma ^{}_{\scriptscriptstyle T}$—a situation in which Rouder and Haaf (2018) acknowledged that large $N_P$ would be especially helpful. For both of these reasons, it is important for future researchers to consider practice-related changes when undertaking power calculations.

As was mentioned in the introduction, Brysbaert and Stevens (2018) used a similar approach to see how the power of more complex LME analyses depends on the numbers of participants and trials in psycholinguistic studies with both participants and items as random factors. With LME models, power depends on the variance between items as well as that between participants (e.g., Westfall et al., 2014). Brysbaert and Stevens (2018) used simulations to examine the power of the LME model to detect orthographic priming effects with different numbers of participants and trials—for example, within the dataset of Adelman et al. (2014). They varied both $N_P$ and $N_T$ and sought to determine what values were needed to achieve adequate power (i.e., 80%) to detect the small priming effect (16 ms effect) that was present in the full dataset. Rather than using the first $N_T$ trials from each participant, however, they used a random selection of $N_T$ trial from each participant like Baker et al. (2021), thus also ignoring any practice effects that might have been present^8^. As expected, they found that power increased with both $N_P$ and $N_T$—holding the other one constant—and that a total of approximately 1,600 RTs per condition were needed to have adequate power. Interestingly, power was not much influenced by the particular $[N_P,N_T]$ combination used to obtain that total number of trials.

The present simulations were intended to inform researchers planning RT experiments whose analyses include a single random participants factor (e.g., *t*-tests), which is a simpler situation than the one considered by Brysbaert and Stevens (2018). With *t*-tests, participants are considered to be the only random factor, and all trial-to-trial RT variation is attributed to pure random variability rather than item effects. Unfortunately, only a few large datasets without item effects could be found for the present simulations, because most existing large datasets come from psycholinguistic studies in which item effects are present. Thus, the present *t*-test-based simulations with these psycholinguistic datasets essentially ignored item effects and treated all trial-to-trial RT variability as random. It should be emphasized that this simplification was a purely heuristic maneuver made in the interests of conducting simulations with large real RT datasets having realistic variation among participants, realistic practice effects, and so on. The present simulations of *t*-tests with psycholinguistic datasets are not meant as a suggestion that item effects can be ignored—a practice which has long been known to be statistically inappropriate (e.g., Clark, 1973).

Because the psycholinguistic datasets used in most of the present simulations also included a random “items” factor—that is, they included trial-to-trial RT variation that could be systematically attributed to differences among items—it is important to consider the likely effect of this variance on the present conclusions. With respect to *t*-test analyses, this item variance would artificially inflate the apparent trial-to-trial RT variability, $\sigma ^{}_{\scriptscriptstyle T}$. Thus, it is reasonable to consider how the $N_P$ versus $N_T$ trade-off observed in simulations with these psycholinguistic datasets would have been different if the datasets had had smaller $\sigma ^{}_{\scriptscriptstyle T}$ values without such item variance. The answer can be seen in Equation 1. As $\sigma ^{}_{\scriptscriptstyle T}$ gets smaller, $N_T$ has a smaller influence on $\sigma ^{}_{\scriptscriptstyle \hat{\Delta }}$; in the extreme with $\sigma ^{}_{\scriptscriptstyle T}=0$, for example, $N_T$ has no effect at all. Thus, as $\sigma ^{}_{\scriptscriptstyle T}$ gets smaller, it becomes more important to have a large $N_P$ rather than a large $N_T$. Without item differences inflating trial-to-trial RT variance in the present simulations, then, it is likely that optimal $[N_P,N_T]$ combinations would involve even larger $N_P$ values and correspondingly smaller $N_T$ values than those suggested by the present simulations with the psycholinguistic datasets. In short, the power advantage associated with large $N_P$ will tend to be stronger in RT paradigms lacking item variance.

The current simulation approach could also be used to study the optimal $[N_P,N_T]$ combinations for detecting myriad other types of effects as well as the difference between two condition mean RTs examined here. For example, RT researchers might look for mean RT differences among three or more conditions, for linear trends across the levels of some independent variable, for two-factor (or higher) interactions, or for condition effects on parameter estimates within a particular RT model. Practice-related changes in any of these types of effects would surely influence the optimal $[N_P,N_T]$ trade-off point, and such practice-related changes could be assessed within analogous simulations using suitable datasets. Of course, it would probably not be cost-effective to collect massive datasets solely to study practice-related changes in any of these other types of effects. Nonetheless, the present results suggest that practice-related changes in observed effect sizes—or the lack thereof—should routinely be described to facilitate the planning of high-powered follow-up studies.

Finally, in some situations constraints imposed by the research questions or setting may dictate the choices of $N_P$ and $N_T$, in which case power considerations are moot. For example, Mazor and Fleming (2022) sought to examine the presence of a certain effect early in practice. This study necessarily used a small $N_T$, because only the initial trials from each participant were relevant to the researchers’ questions, and this implied that a large $N_P$ would be needed to get stable results. Alternatively, researchers might be interested in an effect size at asymptotic practice levels, in which case a large $N_T$ would be essential. A large $N_T$ would also be needed in studies of effects that take some time to develop and can therefore only be assessed after a certain amount of practice (e.g., probability or learning effects). In the absence of such design-based constraints or other indications that the effects under study differ importantly from the effects in the present real datasets, though, the suggestion from these analyses of real datasets is that power is more likely to be maximized with a large number of participants than with a large number of trials per participant.

## Figures and Tables

**Fig. 10:**
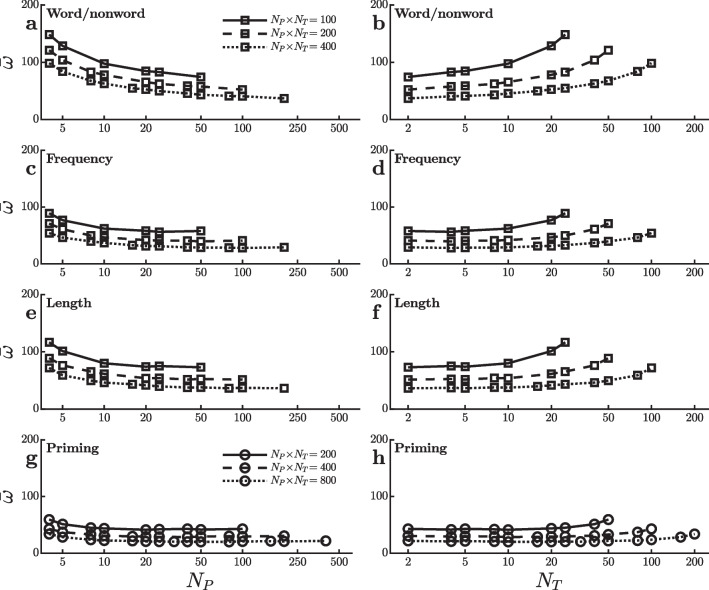
Mean width of confidence intervals for effect size ($\bar{\omega }$) for the virtual studies shown in Fig. 1

**Fig. 11:**
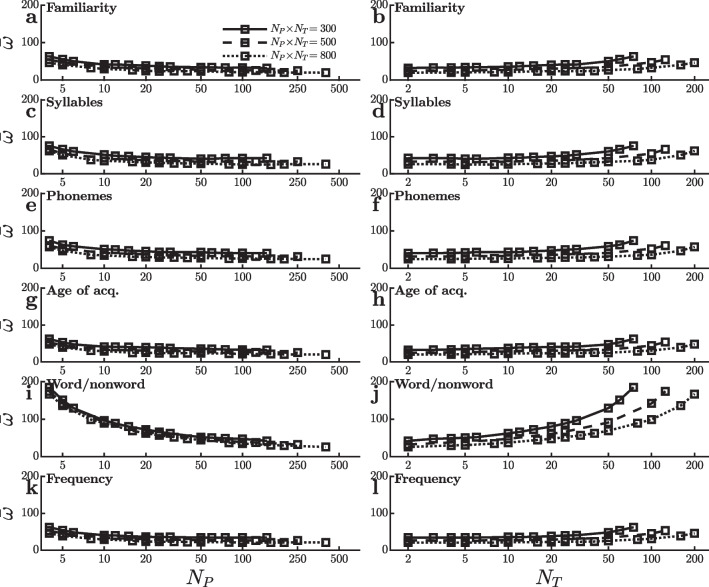
Mean width of confidence intervals for effect size ($\bar{\omega }$) for the virtual studies shown in Fig. 3

**Fig. 12:**
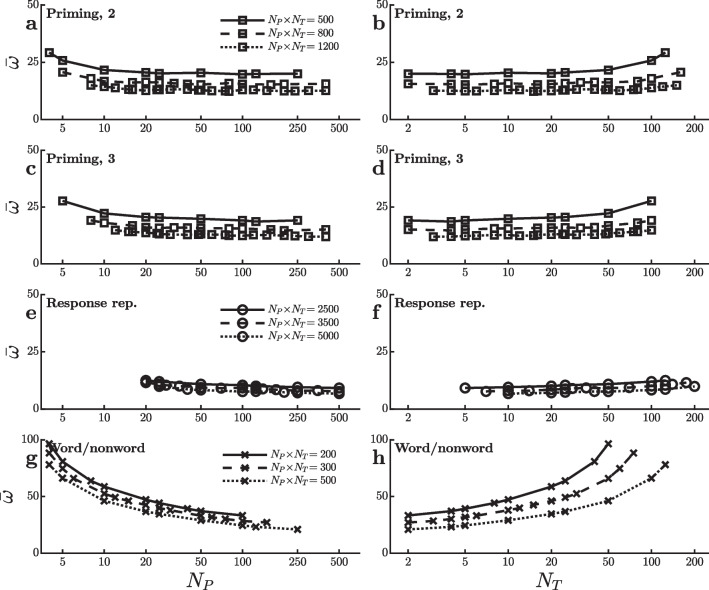
Mean width of confidence intervals for effect size ($\bar{\omega }$) for the virtual studies shown in Fig. 5

**Fig. 13:**
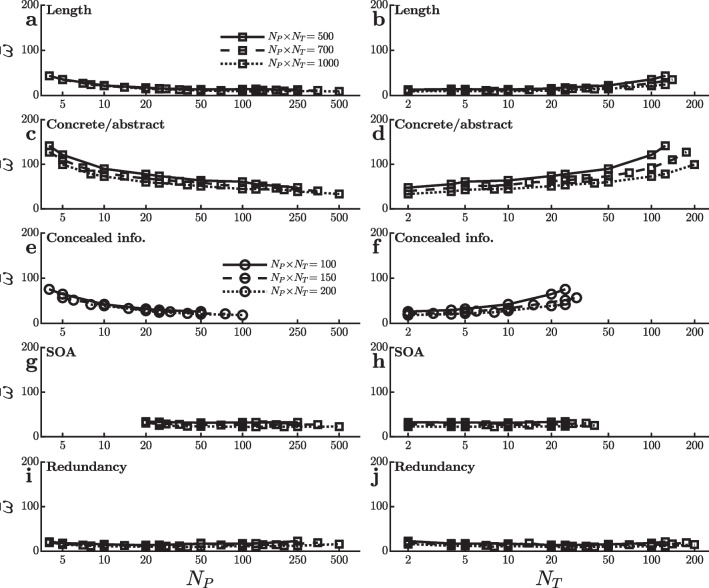
Mean width of confidence intervals for effect size ($\bar{\omega }$) for the virtual studies shown in Fig. 7

**Fig. 14:**
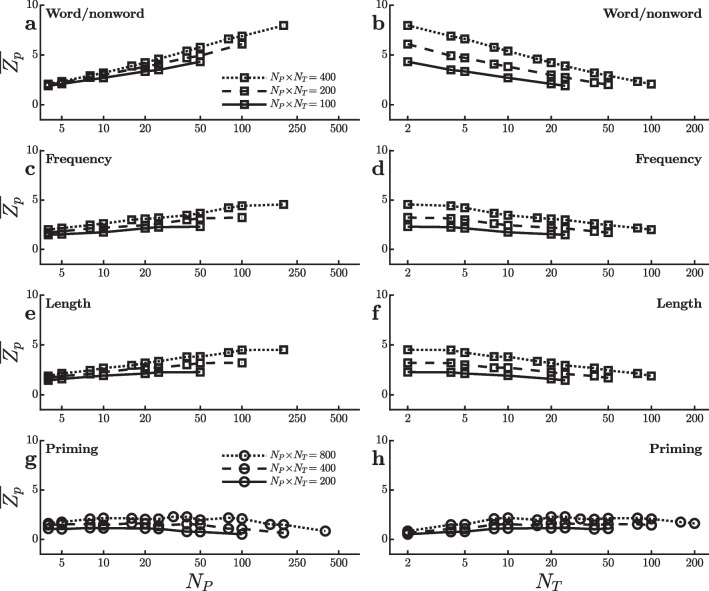
Mean $\overline{Z_p}$ for an effect on mean reaction time (RT) for the virtual studies shown in Fig. 1

**Fig. 15:**
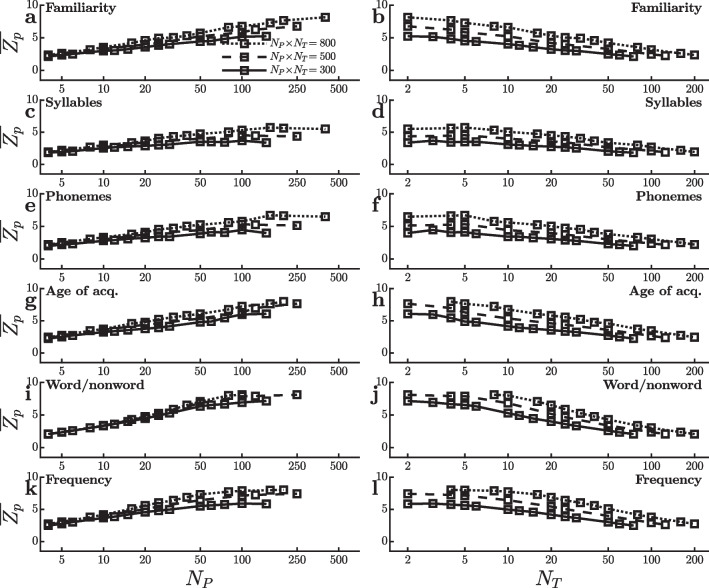
Mean $\overline{Z_p}$ for an effect on mean reaction time (RT) for the virtual studies shown in Fig. 3

**Fig. 16:**
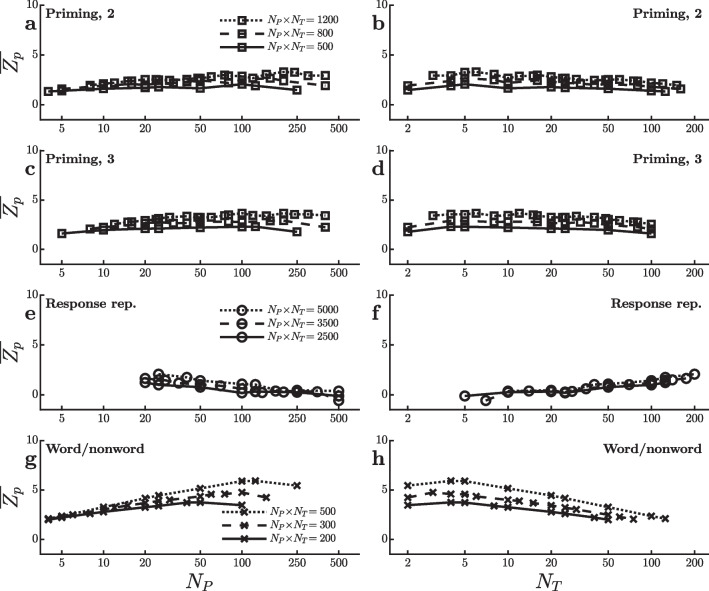
Mean $\overline{Z_p}$ for an effect on mean reaction time (RT) for the virtual studies shown in Fig. 5

**Fig. 17:**
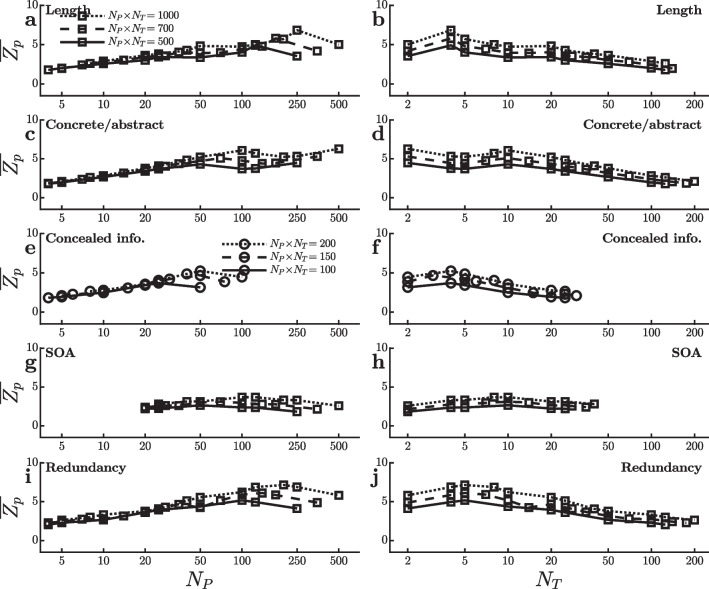
Mean $\overline{Z_p}$ for an effect on mean reaction time (RT) for the virtual studies shown in Fig. 7

## Data Availability

The megastudy datasets were retrieved from the on-line repositories indicated in the original publications of Adelman et al. (2014); Bazilinskyy and De Winter (2018); Goh et al. (2020); Hutchison et al. (2013); Lubczyk et al. (2022); Miguel-Abella et al. (2022), and Pexman et al. (2017). The dataset used for the simulations with the orthographic priming effects in the dataset of Adelman et al. (2014) was that provided by Brysbaert and Stevens (2018).

## Appendix

### Additional Results of Virtual Experiments

This appendix presents figures depicting additional results of the simulated virtual experiments for which power, effect size, and effect variability values are displayed in the figures of the main text.

Figures 10, 11, 12, and 13 show the average confidence interval widths ($\bar{\omega }$) for the effects considered in Figs. 1, 3, 5, and 7, respectively. These were obtained by computing the width, $\omega$, of a 95% *t* confidence interval for the size of the experimental effect in each simulated virtual experiment. These width values were then averaged across experiments to obtain the plotted values of $\bar{\omega }$. Smaller values of $\bar{\omega }$ indicate greater power to detect an effect of a given size, and these interval widths are less subject to ceiling effects than power and would thus allow comparisons among [$N_P$,$N_T$] combinations even with essentially perfect power. The strongest trend across these figures is that mean confidence interval widths generally decrease with increases in the number of participants, consistent with the trends in power. This trend was even present with the semantic priming effect (Fig. 10g), the response repetition effect (Fig. 12e), and the irrelevant versus probe nontarget effect (Fig. 13e), despite the fact that power had dropped for the largest values of $N_P$ in these cases (Figs. 1g and 7e). As mentioned in the main text, those power losses at large $N_P$ seemed to be due at least partly to the small effect sizes early in practice (Figs. 2g, 6e, and 8e), but—unlike power—confidence interval widths are independent of effect size.

Figures 14, 15, 16, and 17 show an additional power-related measure computed for the virtual studies summarized in Figs. 1, 3, 5, and 7, respectively. This measure, $\overline{Z_p}$, is the average across virtual experiments of $Z_p=\Phi ^{-1}(1-p/2)$, where $\Phi$ is the cumulative distribution function of the standard normal distribution (e.g., $Z_p=1.96$ for a virtual experiment yielding an observed $p=0.05$). Thus, larger values of $Z_p$ indicate stronger effects and thus suggest greater power. Like confidence interval widths, $\overline{Z_p}$ values are not subject to ceiling effects, because $Z_p$ values can continue increasing beyond the critical *p* level, and these values would also allow more fine-grained comparisons of conditions with essentially perfect power. Unlike confidence interval widths, $\overline{Z_p}$ is sensitive to effect size as well as variability and is therefore more directly related to power for measuring actual effects than is $\bar{\omega }$. The main message of Figs. 14, 15, 16, and 17 is that the $\overline{Z_p}$ values track the power values rather well, with little or no sign that any sensitivity changes have been obscured by ceiling effects within the power analyses.

## Footnotes

1 An anonymous reviewer asked why the effect of semantic priming might increase with practice, in contrast to the decrease seen with the other effects. One possibility is that the semantic priming effect is partly driven by strategic factors, as is also suggested by sensitivity of the priming effect to the overall proportion of semantically-related prime-target pairs in a study (e.g., Den Heyer et al., 1983; De Wit & Kinoshita,  2015). The influence of relatedness proportion suggests that participants’ word recognition systems somehow become adapted to the nonrandomness of the stimulus sequence (e.g., successive word pairs are semantically related more often than would happen by chance). Such adaptation could allow the systems to take advantage of semantic relatedness to speed responses, but it would need some trials (i.e., practice) for the nonrandomness to be detected and for the adaptation to take place.

2 To obtain the plotted overall $s^{}_{\scriptscriptstyle \hat{\Delta }}$ values, the standard error of the estimated effect was computed with the usual formula for each virtual experiment (i.e., standard deviation of individual-participant difference scores divided by square root of $N_P$). The average of the squares of these standard errors was then computed across virtual experiments, and the plotted $s^{}_{\scriptscriptstyle \hat{\Delta }}$ values are the root mean squares of these values across experiments. These are estimates of the true standard errors of the effect sizes for each effect measured with each [$N_P$,$N_T$] combination.

3 In practice it is more complicated than this because both $\sigma ^{}_{\scriptscriptstyle T}$ and $N_T$ might vary across participants and conditions—the latter especially if researchers eliminate errors or trials with RTs identified as outliers. Detailed consideration of the effects of such further variation on power are beyond the scope of the present analysis.

4 The word-trial RTs and priming classifications for these simulations were those in the datasets of Brysbaert and Stevens (2018) at https://osf.io/fhrc6.

5 This is the dataset of Adelman et al. (2014) at https://files.warwick.ac.uk/jadelman2/browse#FPP, which includes both word and nonword trials. All participants were included in the analysis, regardless of the list counterbalancing used to select participants for analysis in the original study.

6 The CIT test data analyzed here were collected following three practice blocks included to make sure that participants correctly discriminated familiar versus unfamiliar items.

7 For each effect in each of the full datasets, an ANOVA was conducted to check for an interaction of the effect with practice. These ANOVAs yielded interactions with $p<0.001$ for five of the effects, $0.001<p<0.05$ for six of them, $0.05<p<0.1$ for three, and $p>0.1$ for five.

8 Figure 6a–d suggest that these practice effects were small, and in fact they were not significant in the ANOVAs mentioned in footnote 7. Thus, the results of Brysbaert and Stevens (2018) were probably not affected much by ignoring these effects.

## Code Availability

All computations and simulation were carried out using MATLAB. Most of the underlying general-purpose code is available at https://github.com/milleratotago/RawRT and https://github.com/milleratotago/Cupid, and other special-purposes routines are available by request from the author.
